# WTAP‐Mediated m6A Modification of TXNDC5 mRNA Promotes Cervical Carcinogenesis

**DOI:** 10.1002/kjm2.70259

**Published:** 2026-08-14

**Authors:** Ling Chen, Nuo‐Xin Hu, Zhao‐Ying Chen, Ke‐An Zhu, Wei Huang

**Affiliations:** ^1^ Department of Gynecology Hunan Provincial People's Hospital (The First‐Affiliated Hospital of Hunan Normal University) Changsha Hunan China; ^2^ Department of Obstetrics and Gynecology Hunan Normal University Changsha Hunan China

**Keywords:** cervical cancer, N6‐methyladenosine, TXNDC5, WTAP

## Abstract

WT1‐associated protein (WTAP), a core component of the methyltransferase complex, is involved in various tumor pathological processes, but its specific mechanism in cervical cancer (CC) remains unclear. This study, based on single‐cell transcriptomic data (including 3 CC and 2 normal tissues), constructed a CC microenvironment cell atlas through unsupervised clustering and identified a novel malignant subpopulation, TXNDC5^+^ epithelial cells (TXNDC5^+^EPCs). This epithelial subpopulation was specifically enriched in cancerous tissues compared to normal tissues. Furthermore, within the TXNDC5^+^EPC subpopulation, WTAP and TXNDC5 were co‐expressed. In vitro experiments demonstrated that knocking down WTAP reduced the m6A modification level, mRNA stability, and expression of TXNDC5. RIP experiments confirmed their direct binding. In CCK‐8, colony formation, Transwell assays, flow cytometry, and Western Blot analysis, WTAP knockdown inhibited cell proliferation/migration and accelerated apoptosis, while TXNDC5 overexpression reversed these effects. This study is the first to elucidate that the TXNDC5^+^EPC subpopulation is a dominant malignant driver in CC, regulated by WTAP‐mediated m6A post‐transcriptional modification. Targeting the WTAP‐TXNDC5 axis holds promise as a novel therapeutic strategy for CC, directing a new pathway for clinical intervention.

## 1 Introduction

Cervical cancer (CC) is one of the most prevalent malignancies among females worldwide, with approximately 340,000 deaths and 600,000 new cases annually [1]. Although HPV vaccine promotion and advances in early screening techniques have reduced CC incidence, treating advanced or recurrent patients remains a huge challenge [2], necessitating in‐depth exploration of its molecular mechanisms to develop new therapeutic strategies. In recent years, RNA epigenetic modifications, particularly N6‐methyladenosine (m6A) modification, have become a research focus in tumorigenesis and development. m6A modification is the most prevalent internal chemical modification in eukaryotic mRNA, co‐regulated by methyltransferase complexes (Writers, e.g., METTL3/METTL14/WTAP), demethylases (Erasers, e.g., FTO, ALKBH5), and recognition proteins (Readers, e.g., the YTHDF family), influencing mRNA splicing, stability, translation, and degradation [3]. Studies indicate that aberrant m6A modification is involved in cell proliferation, invasion, and migration during cancer progression [4, 5, 6], but its specific regulatory mechanisms in CC require further elucidation.

WT1‐associated protein (WTAP) is a core component of the m6A complex, including METTL3 and METTL14. Although it lacks direct catalytic activity, it is crucial for maintaining complex stability, localization to nuclear speckles, and catalyzing m6A methylation of substrate mRNAs [7]. WTAP expression is dysregulated in various solid tumors (e.g., esophageal squamous cell carcinoma, colorectal cancer, and prostate cancer) and promotes malignant biological behaviors of tumor cells by regulating the m6A modification levels of downstream target genes [8, 9, 10]. However, the specific function of WTAP in CC and its downstream targets and molecular mechanisms remain to be fully clarified.

Increasing evidence suggests that tumor cells hijack the UPR signaling pathway to suppress endoplasmic reticulum (ER) stress‐induced apoptosis, thereby surviving and proliferating in the harsh tumor microenvironment, which has become an important target for cancer therapy [11, 12]. Thioredoxin domain‐containing protein 5 (TXNDC5) is a pivotal ER chaperone protein belonging to the protein disulfide isomerase family, involved in maintaining protein folding homeostasis and redox balance [13]. In recent studies, TXNDC5 is aberrantly highly expressed in various cancers, including CC, and drives tumor proliferation and metastasis by inhibiting excessive ER stress [14, 15]. Notably, TXNDC5 mRNA expression is regulated by m6A modification [15], suggesting it may be a key downstream effector molecule of m6A methyltransferases. Thus, in‐depth research into the mechanism of TXNDC5 in tumors, especially its relationship with m6A modification, is crucial for interpreting the molecular mechanisms of tumorigenesis and development.

This study thoroughly investigates the mechanism of WTAP in CC, revealing its impact on the malignant behavior of CC cells by regulating TXNDC5 m6A modification. These findings provide a theoretical basis for developing WTAP‐TXNDC5 axis‐targeted therapies, potentially offering new approaches and potential targets for clinical intervention in CC.

## 2 Materials and Methods

### 2.1 Single‐Cell Transcriptome Data Acquisition

The single‐cell transcriptome data applied in this study were sourced from the GSE208653 dataset in the public GEO database. This dataset includes single‐cell RNA sequencing data from 3 CC patients and 2 Healthy Individual (HI) cervical tissues.

### 2.2 Single‐Cell Transcriptome Data Analysis

The “Seurat” package was applied for quality control (excluding cells with mitochondrial genes > 20% or gene count < 200), standardization, and batch correction. PCA dimensionality reduction and graph clustering were implemented based on highly variable genes to identify major cell types. The epithelial cell (EPC) subpopulation was extracted from the total cell population for in‐depth analysis. The inferCNV algorithm was utilized to analyze copy number variations (CNVs) in TXNDC5^+^EPCs and other epithelial subpopulations to infer malignancy. GO/KEGG enrichment analysis was constructed on markedly upregulated genes in TXNDC5^+^EPCs to reveal their potential biological functions.

### 2.3 Cell Culture

CC cell lines (HeLa, SiHa, CaSki) (bluefcell, China) and the normal cervical EPC line H8 (bluefcell, China) were cultured in DMEM complete medium (Cellmax, China) supplemented with 10% FBS and 1% PS at 37°C with 5% CO_2_. Cells were maintained by refreshing the medium every 1–2 days and subculturing at 90% confluency.

### 2.4 Cell Transfection

Small interfering RNA (siRNA) targeting WTAP (si‐WTAP) and its negative control (si‐NC), as well as the overexpression plasmid pcDNA3.1‐TXNDC5 (oe‐TXNDC5) and its corresponding empty vector control (oe‐NC), were sourced commercially from GenePharma (China). These plasmids and siRNAs were transfected into cells utilizing Lipofectamine 3000 (Invitrogen, USA). Cells were collected 48 h post‐transfection to determine transfection efficiency or for subsequent analysis.

### 2.5 Collection of Clinical Specimens

A total of 10 paired tumor tissues and matched adjacent non‐tumor tissues were enrolled in this study, which were histopathologically confirmed from CC patients between September 2025 and March 2026. All specimen collection procedures were conducted in strict accordance with the ethical approval issued by the Ethics Committee of Hunan Provincial People's Hospital (The First‐Affiliated Hospital of Hunan Normal University), approval No.: [2026]‐212, and written informed consent was obtained from all enrolled patients prior to sample acquisition.

### 2.6 Immunohistochemistry (IHC)

Serial paraffin sections were prepared from all collected tumor tissues for subsequent IHC staining. Briefly, after deparaffinization and rehydration, tissue sections were subjected to antigen retrieval using Tris‐EDTA buffer (Beyotime, China) for 20 min. Endogenous peroxidase activity was blocked by incubation with 3% hydrogen peroxide solution at room temperature for 10 min. Afterwards, sections were sealed with 10% goat serum (Beyotime, China) for 30 min at ambient temperature. Subsequently, primary antibodies against TXNDC5 (19834–1‐AP, Proteintech, USA) and WTAP (ab195380, Abcam, UK) were applied to the sections and incubated overnight at 4°C. On the following day, sections were incubated with goat anti‐rabbit IgG secondary antibody (ab6721, Abcam, UK) for 1 h at room temperature. Visualization was performed via 5 min incubation with diaminobenzidine (DAB, Yeasen Biotechnology, China), followed by hematoxylin counterstaining (Gene Tech, China) for 1 min. After staining completion, tissues were dehydrated and cleared with gradient ethanol and xylene, and finally mounted with neutral balsam. Five random visual fields were selected and observed under a light microscope (Carl Zeiss, Germany) at a magnification of 400×. The IHC staining intensity (*I*) was graded as 0 (negative), 1 (weak), 2 (moderate), and 3 (strong). The final IHC score was calculated by multiplying the percentage of positive cells (*P*) by staining intensity (*I*), following the formula *Q* = *P* × I. All specimens were stratified into WTAP high‐expression and low‐expression groups based on the median cutoff value.

### 2.7 qPCR

Total RNA was extracted by implementing TRIzol reagent (Invitrogen, USA), followed by reverse transcription with the PrimeScript RT reagent Kit (Takara, Japan). Each sample was set up with three replicate wells, and quantitative PCR was performed in triplicate wells for each sample using the TB Green Premix Ex Taq II (Tli RNaseH Plus) kit (Takara, Japan). The 20 μL reaction system underwent amplification under the following conditions: 95°C pre‐denaturation for 30 s; 40 cycles of 95°C denaturation for 5 s and 60°C annealing for 30 s. The 2^−ΔΔCt^ method was applied to quantify results. Primers for WTAP, TXNDC5, and GAPDH are provided in Table 1.

**TABLE 1:** Primer sequence for qPCR.

Gene	Primer sequence (5′ → 3′)
WTAP	F: CTTCCCAAGAAGGTTCGATTGA
	R: TCAGACTCTCTTAGGCCAGTTAC
TXNDC5	F: AGGTTCGTGGCTATCCCACT
	R: TCCGTCGCTCCAGTCTCTG
GAPDH	F: GGAGCGAGATCCCTCCAAAAT R: GGCTGTTGTCATACTTCTCATGG

### 2.8 Western Blot (WB)

Following protein extraction employing RIPA lysis buffer, protein concentration was determined utilizing a BCA protein assay kit (Beyotime, China). 20 μg of protein was loaded for SDS‐PAGE gel electrophoresis for 80 min and transferred to a PVDF membrane (Millipore, USA). After 1‐h blocking with 5% skim milk, the membrane was subjected to overnight incubation at 4°C with primary antibodies against TXNDC5 (19834‐1‐AP, Proteintech, USA), WTAP (ab195380, Abcam, UK), BAX (ab32503, Abcam, UK), Caspase 3 (ab32351, Abcam, UK), Cleaved‐Caspase 3 (ab32042, Abcam, UK), and GAPDH (ab181602, Abcam, UK), followed by washing. Subsequently, the membrane was treated with secondary antibody IgG (ab205718, Abcam, UK) with gentle shaking at room temperature for 1 h, washed again, incubated with ECL reaction solution, and developed using a chemiluminescence imaging system (CLINX, China).

### 2.9 RIP Assay

The Magna RIP RNA‐Binding Protein Immunoprecipitation Kit (Millipore, USA) was applied for RIP assays. After cell lysis, the lysate was mixed with magnetic beads conjugated with WTAP antibody (ab195380, Abcam, UK) or IgG antibody (3900S, Cell Signaling Technology, USA) and incubated overnight at 4°C. After six washes with pre‐chilled RIP Wash Buffer, RNA‐protein complexes were digested with Proteinase K solution, followed by RNA extraction. Enrichment of TXNDC5 mRNA was quantified by qPCR to validate the interaction between WTAP and TXNDC5 mRNA.

### 2.10 MeRIP‐qPCR

The MeRIP kit (C11051‐1, ribobio, China) was implemented for MeRIP‐qPCR experiments to assess the m6A methylation level. First, 1–2 μg of high‐quality total RNA was sonicated at 4°C to generate 100–500 nt fragments. Subsequently, these fragments underwent a 2‐h incubation at 4°C with m6A antibody (ABE572, Merck Millipore, Germany)‐coated magnetic beads. After thorough washing with low‐salt and high‐salt buffers, RNA complexes were dissociated using an elution buffer containing competitive m6A at 30°C and purified with a Qiagen kit (12123, Germany). Finally, the eluted RNA was detected by qPCR.

### 2.11 mRNA Stability Assay

HeLa cells were treated with actinomycin D (final concentration 5 μg/mL) and harvested at 0, 3, and 6 h post‐treatment. Total RNA was extracted, and TXNDC5 mRNA stability was evaluated via qPCR quantification of its expression levels.

### 2.12 CCK‐8 Assay

CC cells in the logarithmic growth phase were inoculated into 96‐well plates. 10 μL of CCK‐8 reagent (Beyotime, China) was added to each well, followed by 1‐h incubation at 37°C. The optical density value at 450 nm wavelength was captured by employing a microplate reader (Thermo Fisher Scientific, USA) at different time points (0, 24, 48, 72 h), and proliferation curves were plotted.

### 2.13 Colony Formation Assay

Following a 10‐day culture of 1 × 10^3^ cells/well in 6‐well plates (2 mL medium), cells underwent medium removal, PBS rinse, and fixation with 4% paraformaldehyde (30 min). Staining was conducted using 1 mL crystal violet (Beyotime, China) for 20 min, with subsequent washing, air‐drying, and documentation.

### 2.14 Wound Healing Assay

The harvested logarithmic‐phase CC cells from each group were trypsinized and centrifuged. The cell suspension was inoculated into 6‐well plates. At ≥ 95% confluency, a cross was scratched in the center of each well using a white pipette tip perpendicular to the plate, followed by PBS removal of dislodged cells. Images were taken at 0 and 24 h using an inverted microscope, and migration rates were quantified with ImageJ software.

### 2.15 Cell Apoptosis Assay

Cell apoptosis was detected by flow cytometry using an Annexin V‐FITC Apoptosis Detection Kit (C1062S, Beyotime, China). Treated cells were collected, washed with pre‐cooled PBS, and stained with Annexin V‐FITC in the dark at 4°C for 15 min, followed by PI staining. Finally, detection and analysis were performed by leveraging a flow cytometer (Agilent, USA).

### 2.16 Data Analysis

All experiments were performed in triplicate at least. Statistical analysis was constructed in GraphPad Prism 8 (GraphPad Software Inc., USA), employing the *t*‐test for group comparisons, with significance set at *p* < 0.05.

## 3 Results

### 3.1 Construction of the Single‐Cell Atlas

**FIGURE 1:**
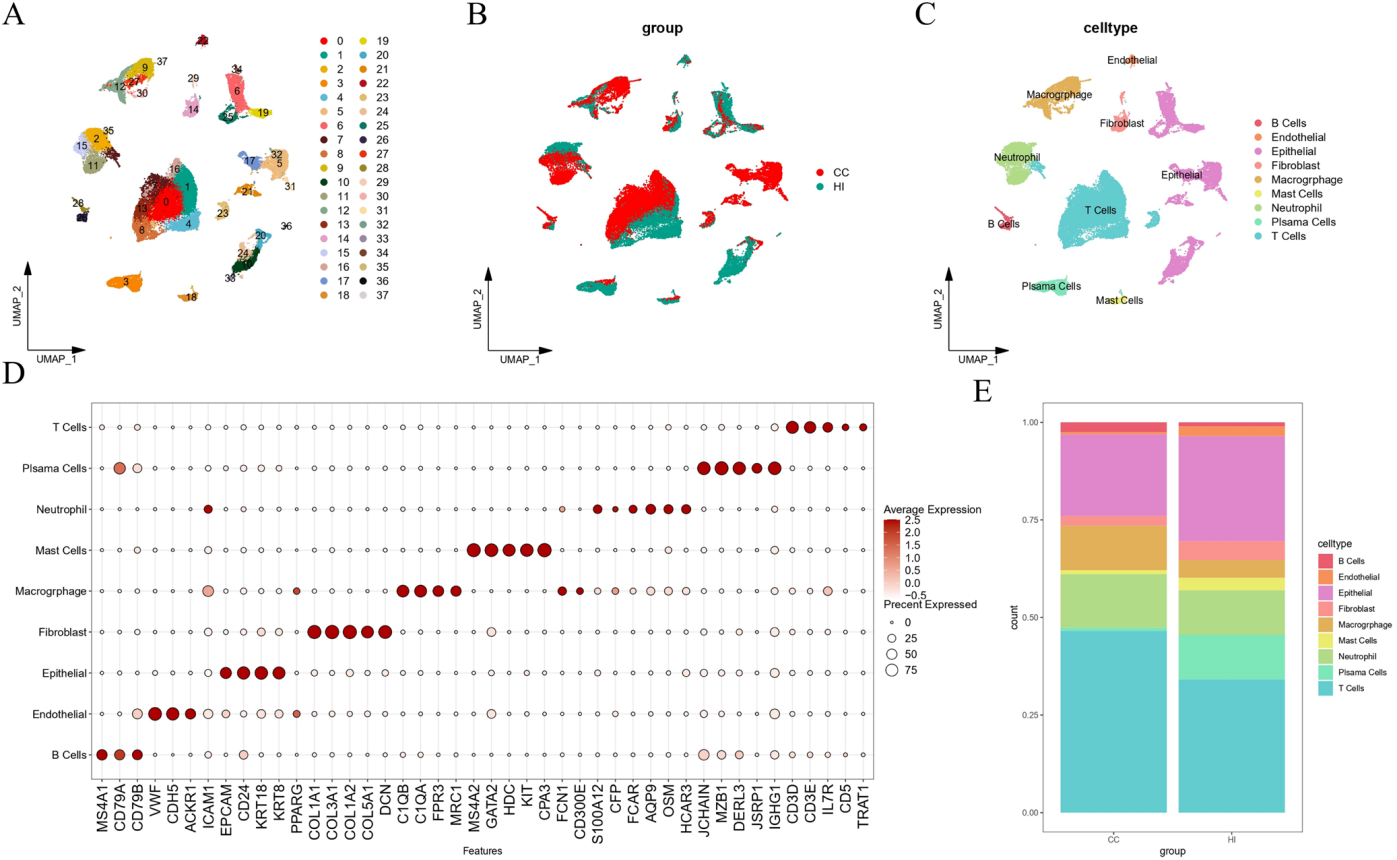
Single‐cell RNA sequencing analysis reveals cell type distribution and characteristic gene expression in cervical cancer and normal tissues. (A) UMAP plot of 38 distinct cell clusters. (B) Distribution of cell clusters in UMAP space based on sample origin. (C) Cell annotation based on unsupervised clustering. (D) Bubble plot of marker genes used for cell annotation. (E) Distribution of different cell types in CC and HI. CC, cervical cancer; HI, normal tissue.

Based on the GEO dataset GSE208653 (3 CC vs. 2 normal controls), high‐quality single‐cell transcriptome data were obtained after quality control, standardization, and batch correction. For UMAP dimensionality reduction technology for cell cluster analysis, 9 major cell subpopulations were identified, including B cells, endothelial cells, EPCs, fibroblasts, macrophages, mast cells, neutrophils, plasma cells, and T cells (Figure 1A–C). The specific expression patterns of classical marker genes in each cell subpopulation were displayed in the bubble plot (Figure 1D), further validating the accuracy of cell type identification. The discrepancies in cellular composition proportions between different samples were compared visually using a stacked bar chart (Figure 1E). Given that EPCs are not only the main structural components of cervical tissue but also the cells of origin for cervical carcinogenesis [16], we subsequently focused on this cell subpopulation for in‐depth molecular mechanism research.

### 3.2 Specific Enrichment of the TXNDC5 ^+^ EPC Subpopulation in Cancer Tissues

**FIGURE 2:**
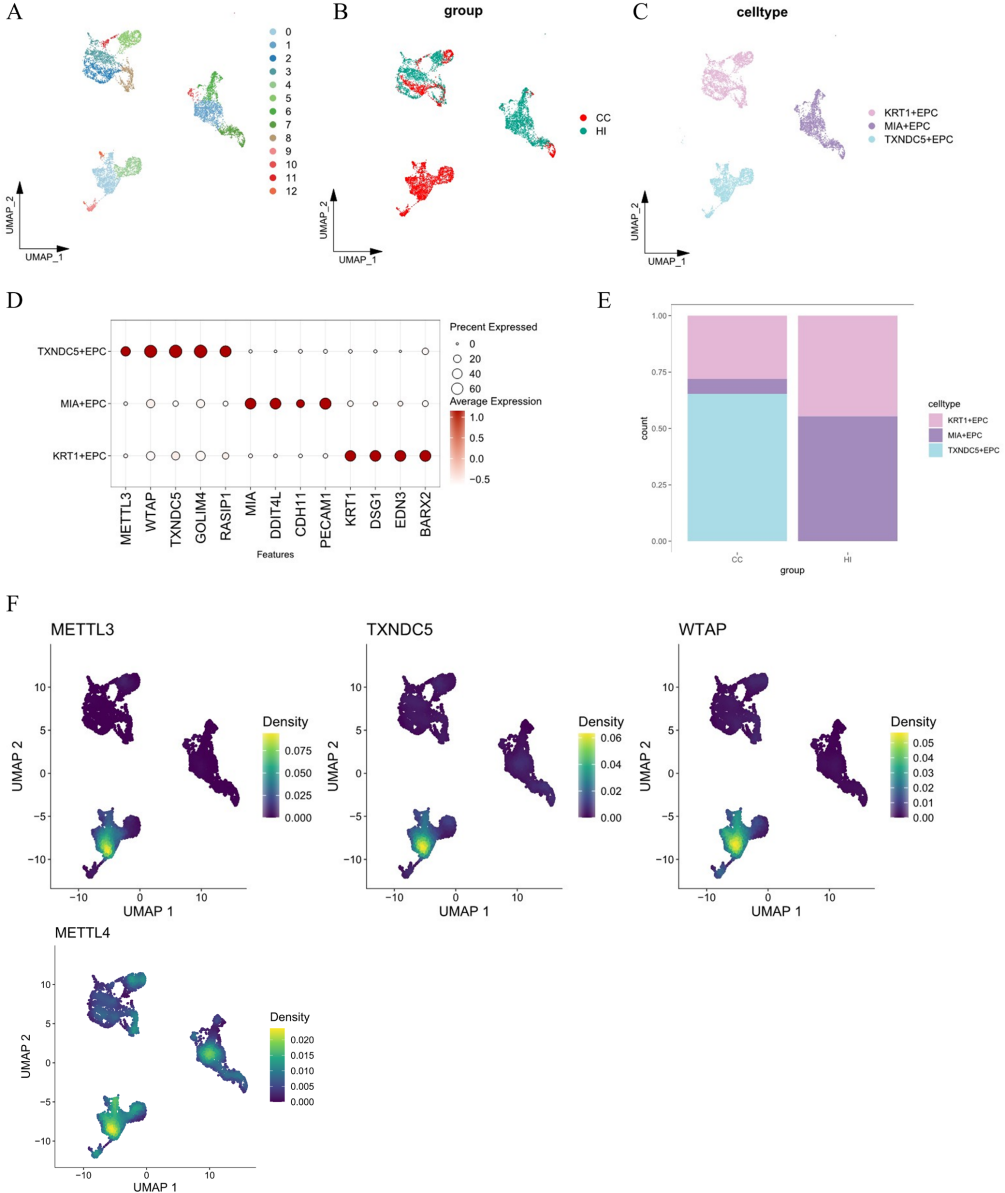
Single‐cell sequencing analysis of epithelial cell subpopulations. (A) UMAP plot of 13 distinct cell clusters. (B) UMAP clustering comparison of cell subpopulations between CC and HI. (C) UMAP plot of different epithelial cell subpopulations: KRT1^+^EPC, MIA^+^EPC, and TXNDC5^+^EPC. (D) Feature gene expression analysis plot for epithelial cell subpopulations. (E) Distribution of epithelial cell subpopulations in CC and HI. (F) Expression patterns of METTL3, TXNDC5, WTAP, and METTL14 genes in UMAP space. CC, cervical cancer; HI, normal tissue.

Further subcluster analyses on EPCs were executed. Utilizing UMAP dimensionality reduction and clustering, we identified three EPC subpopulations: KRT1^+^EPC, MIA^+^EPC, and TXNDC5^+^EPC (Figure 2A–C). In differential expression analysis, by using the “Seurat” package, specific marker genes for each subpopulation were identified (Figure 2D). Comparison of cell proportions demonstrated that the TXNDC5^+^EPC subpopulation accounted for an appreciably higher proportion in CC samples versus normal tissues, suggesting its close association with cervical carcinogenesis (Figure 2E). Furthermore, within the TXNDC5^+^EPC subpopulation, core components of the m6A methyltransferase complex (WTAP, METTL3, METTL14) showed marked co‐expression with TXNDC5 (Figure 2F). This phenomenon suggests that m6A modification may be involved in CC progression by regulating TXNDC5.

### 3.3 inferCNV Confirms the Malignant Nature of TXNDC5 ^+^ EPC

**FIGURE 3:**
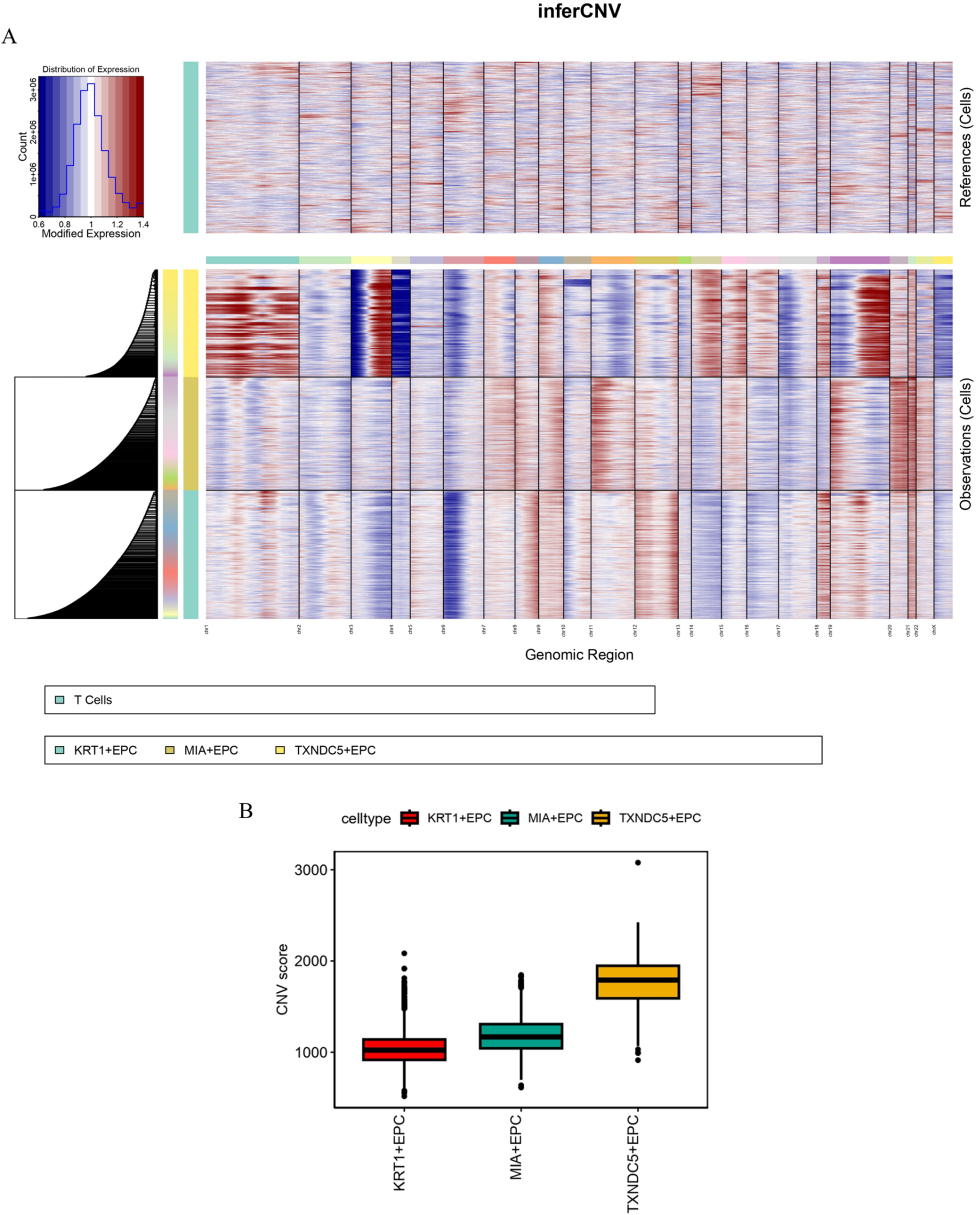
inferCNV confirms the malignant nature of TXNDC5^+^EPC. (A) Copy number variation (CNV) in genomic regions of epithelial cell subpopulations analyzed by inferCNV. (B) CNV scores of epithelial cell subpopulations.

Assessment of malignancy through genome‐wide CNV analysis revealed that the TXNDC5^+^EPC subpopulation exhibited pronounced genomic instability: widespread chromosomal segment amplifications/deletions (Figure 3A). Moreover, the CNV Score of the TXNDC5^+^EPC subpopulation was significantly higher than that of other epithelial subpopulations (e.g., KRT1^+^EPC, MIA^+^EPC) (Figure 3B), thereby confirming that the TXNDC5^+^EPC subpopulation is a malignant tumor EPC phenotype.

### 3.4 Upregulated Genes in TXNDC5 ^+^ EPC Are Enriched in Cancer Pathways

**FIGURE 4:**
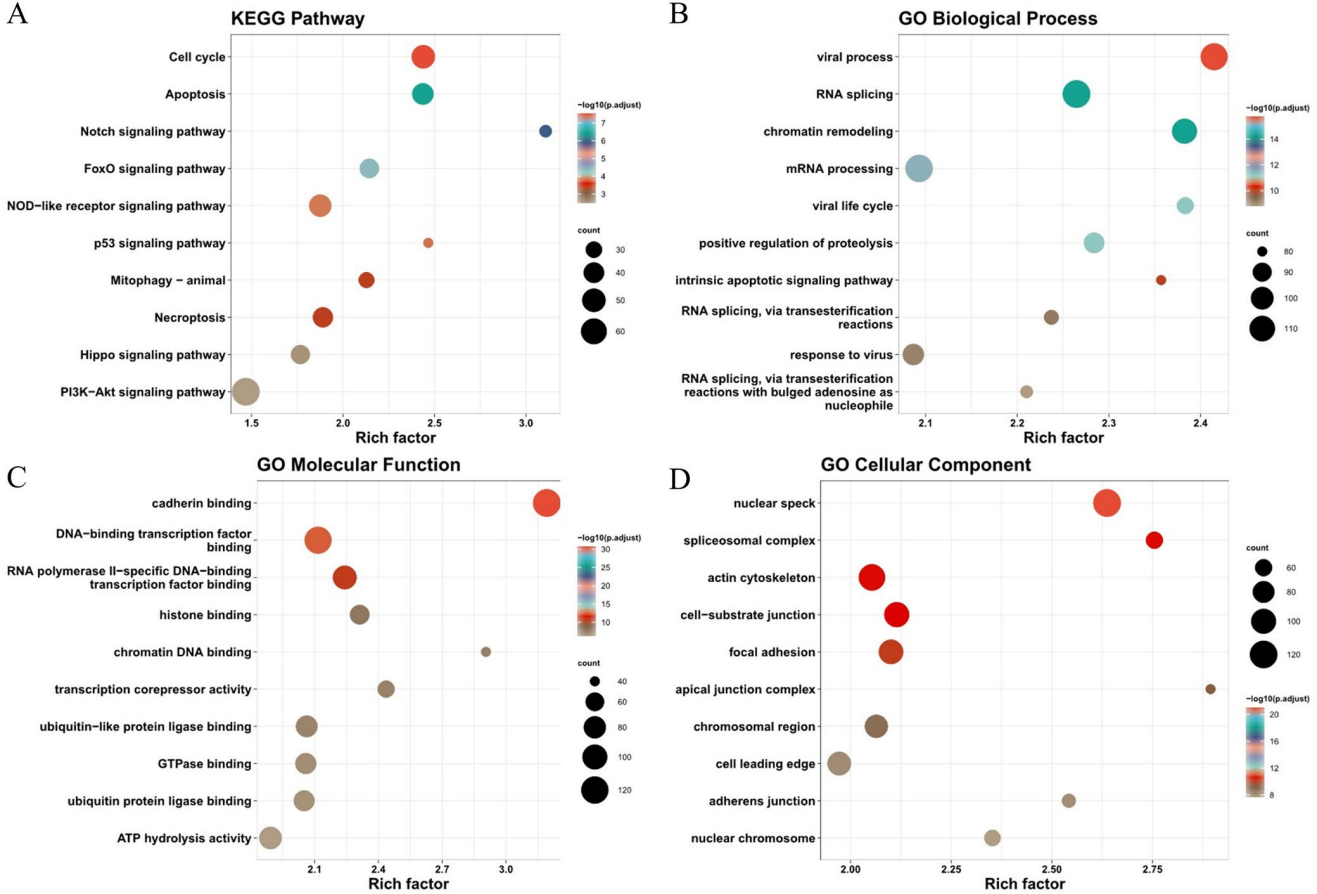
KEGG and GO analyses of upregulated genes in TXNDC5^+^EPC. (A) KEGG pathway enrichment analysis plot. (B) GO biological process enrichment analysis plot. (C) GO molecular function enrichment analysis plot. (D) GO cellular component enrichment analysis plot.

Next, functional enrichment analysis was performed on differentially upregulated genes in the TXNDC5^+^EPC subpopulation compared to other epithelial subpopulations. KEGG pathway analysis showed noticeable enrichment in cell cycle, apoptosis, Notch, and FoxO signaling pathways, suggesting their key regulatory roles in disease progression (Figure 4A). GO functional analysis further revealed that the target gene set drives specific disease phenotypes by coordinating core biological modules such as transcriptional regulation, RNA processing, and cytoskeleton reorganization (Figure 4B–D). These findings indicate that the TXNDC5^+^EPC subpopulation likely holds an essential role in CC occurrence and development.

### 3.5 WTAP Stabilizes TXNDC5 mRNA Through m6A Modification in CC

**FIGURE 5:**
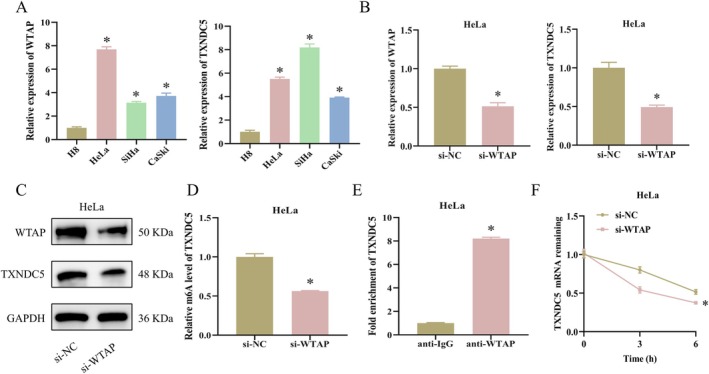
WTAP mediates m6A modification of TXNDC5 in cervical cancer cells. (A) qPCR of WTAP and TXNDC5 expression in cervical cancer cell lines (HeLa, SiHa, CaSki) and normal cervical epithelial cell line (H8). (B, C) qPCR (B) and WB (C) of WTAP and TXNDC5 expression in HeLa cells transfected with si‐NC/si‐WTAP. (D) MeRIP analysis of TXNDC5 m6A modification level. (E) RIP analysis of the interaction between TXNDC5 mRNA and WTAP protein in HeLa cells. (F) qPCR of TXNDC5 mRNA stability in HeLa cell line measured after treatment with 5 μg/mL actinomycin D. *: *p* < 0.05.

To verify the single‐cell sequencing analysis results, we detected the expression of WTAP and TXNDC5 genes in CC cell lines (HeLa, SiHa, CaSki) and the normal cervical EPC line H8 employing qPCR. Both WTAP and TXNDC5 were significantly upregulated in CC cell lines compared to the normal cervical EPC line (Figure 5A). IHC analysis was performed to validate the expression correlation between WTAP and TXNDC5 in human CC tissues. The protein level of TXNDC5 was synchronously downregulated in the WTAP low‐expression group, while it was correspondingly upregulated in the WTAP high‐expression group (Figure S1A). To further investigate the regulatory mechanism of WTAP on TXNDC5, we constructed si‐NC and si‐WTAP transfection systems and transfected them into HeLa cells. To exclude cell line‐specific effects of WTAP knockdown, we further assessed the knockdown efficiency of WTAP in another cervical cancer cell line CaSki using qPCR and WB. The results revealed that both mRNA and protein expression of WTAP were markedly reduced upon WTAP silencing, verifying the successful establishment of the WTAP knockdown cell model (Figure S1B,C). Post‐transfection detection revealed that WTAP knockdown not only significantly reduced the mRNA levels of WTAP and TXNDC5 in HeLa cells (Figure 5B) but also decreased their protein expression (Figure 5C). Furthermore, for the detection of TXNDC5 m6A levels in HeLa cells, WTAP knockdown significantly reduced TXNDC5 m6A levels (Figure 5D), suggesting that WTAP may regulate TXNDC5 expression by mediating m6A modification. To further confirm the interaction between WTAP and TXNDC5 mRNA, we performed RIP assays for WTAP protein in HeLa cells. The results showed that endogenous TXNDC5 mRNA was significantly enriched in WTAP immunoprecipitates compared to the IgG control group (Figure 5E), indicating that WTAP protein can specifically bind to TXNDC5 mRNA. Finally, actinomycin D experiments were leveraged to detect the regulatory effect of WTAP on TXNDC5 mRNA stability. The results showed that WTAP knockdown notably reduced TXNDC5 mRNA stability (Figure 5F), further confirming that WTAP increases TXNDC5 expression levels by facilitating m6A modification‐mediated mRNA stability.

### 3.6 WTAP‐Mediated m6A Modification of TXNDC5 Regulates the Malignant Progression of CC Cells

**FIGURE 6:**
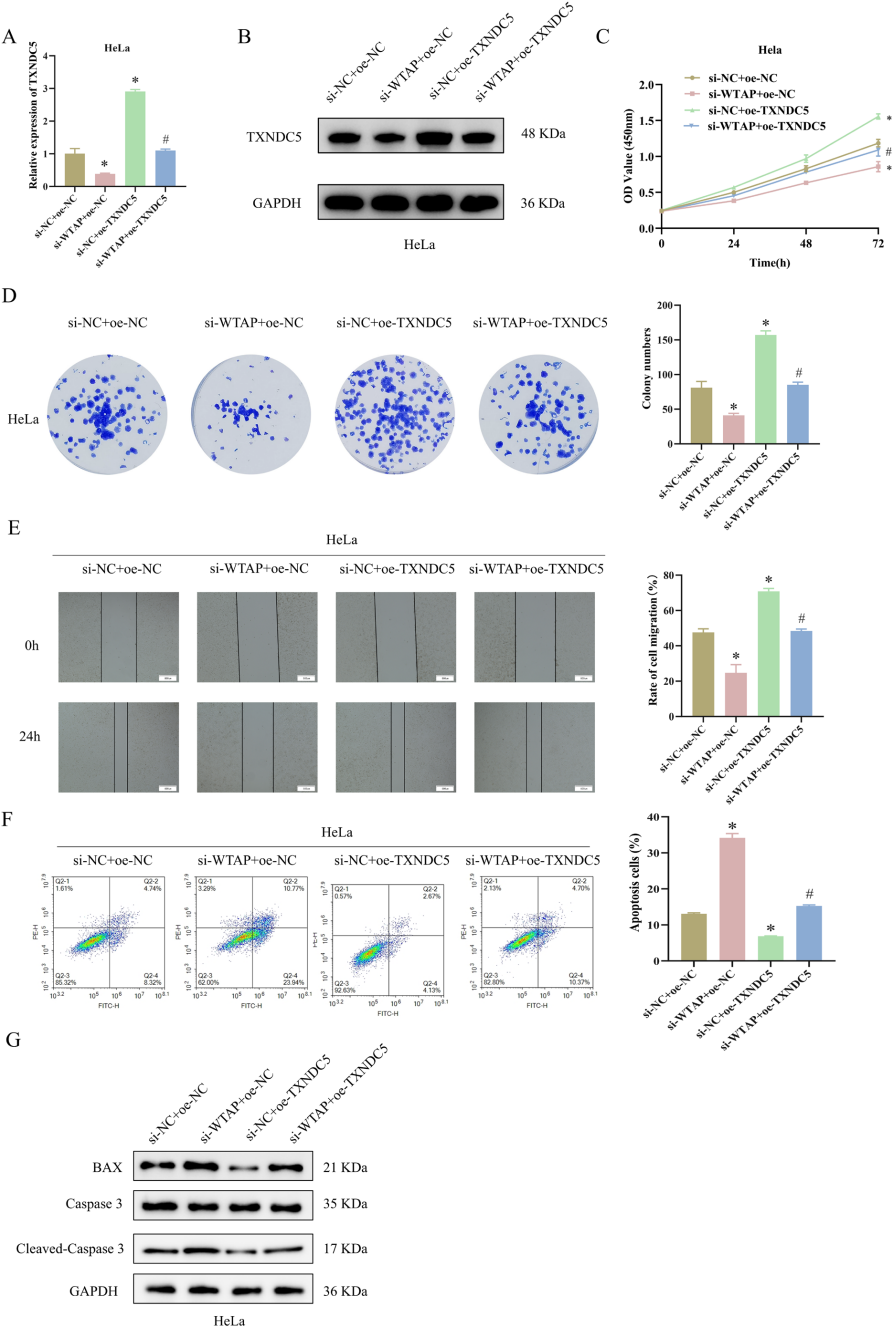
WTAP influences the malignant behavior of cervical cancer cells by regulating TXNDC5. (A, B) qPCR (A) and WB (B) of TXNDC5 expression in HeLa cells transfected with si‐NC + oe‐NC, si‐WTAP+oe‐NC, si‐NC + oe‐TXNDC5, si‐WTAP+oe‐TXNDC5. (C‐D) CCK‐8 (C) and colony formation (D) assays of HeLa cell proliferation ability. (E) Wound healing assay of cell migration ability in different groups. (F) Flow cytometry of cell apoptosis. (G) WB assay of protein expression levels of BAX, Caspase 3, and Cleaved‐Caspase 3. *: *p* < 0.05 vs. si‐NC + oe‐NC. #: *p* < 0.05 vs. si‐WTAP+oe‐NC.

To deeply explore the function of the WTAP‐TXNDC5 axis in CC, this study constructed a co‐transfection system with WTAP knockdown and TXNDC5 overexpression and transfected it into HeLa cells. TXNDC5 expression levels were verified by qPCR and WB experiments (Figure 6A,B). Subsequently, cell proliferation capability was assessed by using CCK‐8 and colony formation assays. The results showed that compared to the control group, WTAP knockdown significantly inhibited CC cell proliferation ability, while TXNDC5 overexpression restored this inhibitory effect to control levels (Figure 6C,D). Furthermore, wound healing assays demonstrated that TXNDC5 overexpression could reverse the inhibitory effect of WTAP knockdown on the migration ability of CC cells (Figure 6E). To further assess the apoptosis level, through flow cytometry, we uncovered that TXNDC5 overexpression significantly inhibited the increase in apoptosis caused by WTAP knockdown (Figure 6F). WB detected the protein levels of apoptosis‐related molecules including BAX, Caspase 3, and Cleaved‐Caspase 3. The results indicated that single WTAP knockdown significantly upregulated the expression of pro‐apoptotic BAX and Cleaved‐Caspase 3 relative to the control group. Notably, TXNDC5 overexpression effectively reversed the upregulation of these pro‐apoptotic proteins induced by WTAP silencing (Figure 6G). The same experimental groups were established in CaSki cells, and qPCR was applied to verify the expression level of TXNDC5 (Figure S2A). Functional assays further demonstrated that TXNDC5 overexpression markedly rescued the suppressed proliferation and impaired migration capacity caused by WTAP knockdown in CaSki cells (Figure S2B–D). In short, oe‐TXNDC5 could rescue the effects of WTAP knockdown in CC cells.

## 4 Discussion

CC, as one of the leading causes of cancer‐related deaths among females globally, involves complex molecular mechanisms and cellular heterogeneity in its pathogenesis and progression [17]. By integrating single‐cell transcriptome sequencing and functional experiments, this study reveals for the first time the molecular mechanism by which WTAP promotes CC progression by regulating TXNDC5 expression through m6A methylation modification. At the single‐cell level, we identified TXNDC5^+^EPC, an EPC subpopulation with remarkable genomic instability. This subpopulation is specifically expanded in CC tissues and exhibits high CNV burden and malignant transformation scores, clearly identifying it as a tumor‐originating cell [18]. Furthermore, KEGG enrichment revealed significant activation of the Notch and FoxO pathways in TXNDC5^+^EPC. This is highly consistent with TXNDC5 acting as a hazard and responding to metabolic and cellular stress signals to promote tumor cell survival [19].

In recent years, research on the role of RNA epigenetic modifications, especially m6A modification, in tumorigenesis has made progress [20]. For example, m6A modification regulated NEK2 mRNA stability in CC, boosting tumor cell growth, migration, and invasion [21]. Through single‐cell transcriptome data analysis, this study found that WTAP is highly expressed in CC EPC subpopulations and shows a co‐expression pattern with METTL3 and TXNDC5. Given that the mechanism by which METTL3 promotes CC cell proliferation and metastasis by regulating TXNDC5 expression has been revealed [15], we hypothesized that WTAP‐mediated m6A modification in TXNDC5 mRNA regulates TXNDC5 levels. In vitro experiments confirmed that WTAP directly binds to TXNDC5 mRNA and reinforces its m6A modification level, thereby stabilizing TXNDC5 mRNA and promoting its expression. This finding not only expands the regulatory network of m6A modification in CC but also provides a new perspective for understanding tumor heterogeneity. Although previous studies have reported that WTAP, as a key component of the m6A methyltransferase complex, assumes a cancer‐promoting role in various cancers [22, 23], its specific regulatory network in CC remains to be elucidated. This study discovered that WTAP knockdown led to decreased TXNDC5 expression, thereby inhibiting CC cell proliferation, migration, and driving apoptosis, while TXNDC5 overexpression reversed these effects. These results establish for the first time a complete chain of evidence for the WTAP‐TXNDC5 regulatory axis in CC and provide a theoretical basis for therapeutic strategies targeting this axis.

Within the m^6^A methyltransferase complex, METTL3 and METTL14 are primarily responsible for catalyzing methylation reactions. As a central regulatory subunit, WTAP exerts its regulatory function mainly by recruiting the catalytic complex to nuclear speckles and directing its recognition of substrate mRNA [24]. Accordingly, although WTAP plays a pivotal role in substrate recognition, METTL3 and METTL14 serve as core catalytic subunits, and their regulatory effects on TXNDC5 should not be overlooked. Previous research has validated that METTL3 modulates the m^6^A modification level of TXNDC5 mRNA, thereby repressing endoplasmic reticulum stress‐triggered apoptosis and facilitating the malignant progression of CC [15]. In addition, METTL3 may regulate TXNDC5 transcription in an m^6^A‐dependent manner, thus participating in the oncogenesis and progression of melanoma [25]. Beyond functioning independently, WTAP may also regulate synergistically the biological behaviors of tumor cells with other m6A modification‐related proteins. Previous studies have shown that METTL3 and WTAP act synergistically in various cancers, co‐regulating the m6A modification levels of downstream genes, thereby affecting tumor cell proliferation, migration, and apoptosis [23, 26]. In this study, we also found co‐expression of WTAP and METTL3 or METTL14 in CC EPC subpopulations, suggesting that they might function through a similar cooperative mechanism in CC. Future research could explore the interactions between WTAP and other m6A‐related proteins and their synergistic regulatory mechanisms in CC, which will help comprehensively understand the role of m6A modification in tumor development.

As a pivotal driver of cervical carcinogenesis, human papillomavirus (HPV) has been proven to be closely correlated with m^6^A modification [27]. Accumulating evidence indicates that HPV oncoproteins participate in the modulation of m^6^A modification through multiple mechanisms. For instance, HPV E7 specifically upregulates the m^6^A demethylase ALKBH5, which reduces the m^6^A modification level of PAK5 mRNA and thereby accelerates CC progression [28]. Hu et al. reported that HPV E6/E7 stabilizes m^6^A‐modified MYC mRNA by regulating IGF2BP2, consequently driving metabolic reprogramming in CC cells [29]. Moreover, the expression levels of METTL3 and WTAP are significantly associated with HPV infection status in CC [30]. In the present study, we observed markedly elevated WTAP expression in HPV‐positive cell lines including HeLa, SiHa and CaSki. This finding suggests that WTAP, as a core regulatory subunit of the m^6^A methyltransferase complex, may be directly or indirectly modulated by viral oncoproteins under HPV infection, and further participates in the malignant progression of CC by altering the m^6^A modification status of TXNDC5. Nevertheless, the regulatory interplay between HPV and the WTAP‐TXNDC5 axis remains to be elucidated in future investigations.

However, this study also has some limitations. First, the sample size for single‐cell sequencing analysis is relatively limited (3 cancer tissues and 2 normal tissues), which may affect the generalizability of the conclusions. Second, the study is primarily based on cell line experiments, lacking animal model validation of the in vivo function of the WTAP‐TXNDC5 axis. Furthermore, whether the expression and functional heterogeneity of the WTAP‐TXNDC5 axis differ across various HPV infection statuses or different pathological types of CC warrants further investigation.

Taken together, this study systematically elucidates for the first time the molecular mechanism by which WTAP‐mediated m6A modification regulates TXNDC5 mRNA stability to promote cervical carcinogenesis and progression, providing a new perspective for deeply understanding the epigenetic regulatory network of CC. These findings deepen our understanding of the critical role of m6A methylation modification in the formation of malignant tumor characteristics. By clarifying the novel oncogenic WTAP‐TXNDC5 axis, it lays a solid theoretical foundation for developing targeted CC treatment strategies based on RNA epigenetic regulation.

## Funding

This work was supported by Hunan Provincial Natural Science Foundation Project (2025JJ80764).

## Ethics Statement

All specimen collection procedures were conducted in strict accordance with the ethical approval issued by the Ethics Committee of Hunan Provincial People's Hospital (The First‐Affiliated Hospital of Hunan Normal University), approval No.: [2026]‐212, and written informed consent was obtained from all enrolled patients prior to sample acquisition.

## Conflicts of Interest

The authors declare no conflicts of interest.

## Supporting information


**Figure S1:** Correlation of WTAP and TXNDC5 expression in CC tissues and validation of WTAP knockdown efficiency in cells. (A) IHC was used to compare the differential protein expression of TXNDC5 between WTAP high‐expression and low‐expression groups in human CC tissues. (B, C) CaSki cells were transfected with si‐NC or si‐WTAP. qPCR and WB were performed to detect WTAP expression for validating the efficiency of the WTAP knockdown cell model.

**Figure S2:** WTAP modulates the malignant phenotypes of CaSki cells via regulating TXNDC5. (A) CaSki cells were transfected with four groups: si‐NC + oe‐NC, si‐WTAP+oe‐NC, si‐NC + oe‐TXNDC5, and si‐WTAP+oe‐TXNDC5. TXNDC5 expression was determined by qPCR. (B, C) CCK‐8 and colony formation assays were conducted to evaluate the proliferative capacity of CaSki cells. (D) Wound healing assay was performed to assess cell migration ability among different groups. *: *p* < 0.05 vs. si‐NC + oe‐NC; #: *p* < 0.05 versus si‐WTAP+oe‐NC.

## Data Availability

The data and materials in the current study are available from the corresponding author on reasonable request.
